# A244 PROVIDING THE FODMAP DIET THROUGH AN ONLINE PLATFORM: IMPACT ON THE QUALITY OF LIFE AND THE PHYSICAL AND PSYCHOLOGICAL SYMPTOMS IN IRRITABLE BOWEL SYNDROME

**DOI:** 10.1093/jcag/gwab049.243

**Published:** 2022-02-21

**Authors:** S Laforce, Y Mukaneza, M Tremblay, B Lamarche, C Delawarde-Saïas, M Bouin, C Bemeur

**Affiliations:** 1 Hepato-Neuro Lab, Centre Hospitalier de l’Universite de Montreal Centre de Recherche, Montreal, QC, Canada; 2 École de nutrition et Institut sur la nutrition et les aliments fonctionnels (INAF), Quebec, QC, Canada; 3 Centre Hospitalier de l’Universite de Montreal, Montreal, QC, Canada; 4 Nutrition, Université de Montréal, Montreal, QC, Canada

## Abstract

**Background:**

Irritable bowel syndrome (IBS) is a functional gastrointestinal disorder that affects approximately 15 % of the worldwide population. It is characterized by recurring abdominal pain and changes in bowel habits. This population is more at risk of suffering from chronic fatigue and psychological distress. Moreover, IBS significantly reduces the quality of life of patients and increases the economic burden on the health system. Many therapies have been developed to try to alleviate the symptoms and improve the lives of people with IBS. Among these is the FODMAP ( *Fermentable Oligosaccharides Disaccharides Monosaccharides and Polyols*) diet which aims to establish a personal tolerance to these nutrients (carbohydrates) that accentuate the symptoms related to IBS. This diet, lasting on average twelve weeks, is separated into three steps: elimination, reintroduction, and personalization. An innovative approach has been developed by the online platform SOSCuisine.com® to allow people with IBS to safely follow the FODMAP diet in a ‘self-service’ way. It consists of using an online service of personalized weekly menus low in FODMAP with tips and instructions for each step of the diet in combination with access to a peer support group, moderated by a specialized registered dietician.

**Aims:**

The aim of this project is to evaluate the impact of this new online service on the quality of life and the control of the physiological and psychological symptoms of people living with IBS. We hypothesized that this online service improve the quality of life and the control of the symptoms.

**Methods:**

The participant has to be at least 18 years old, understand French, have access to Internet and a Facebook account (sufficient familiarity to use them) and have an IBS diagnosis (Rome IV criteria) validated or re-validated by a gastroenterologist within three months. People with eating disorders, mental health problems, other chronic gastrointestinal illnesses (except gastroesophageal reflux disease), diabetes and with a pregnancy are excluded. Validated questionnaires are used to assess the quality of life ( *IBS-Quality Of Life*), physical symptoms ( *IBS-Severity Scoring System*), state and trait ( *State-Trait Anxiety Inventory, Form Y*) whereas food intake is assessed with a online 24-hour dietary recall.

**Results:**

So far, 35 participants have been included in the study (83% female, 45,3±12,8 years old). After the intervention, the severity of symptoms significantly decreased (p<0.001), and quality of life (p=0.041) and state anxiety (p=0.034) significantly improved.

**Conclusions:**

This study should allow the identification of facilitating elements to adhere to the FODMAP diet and possibly improve the quality of life of this population.

**Funding Agencies:**

None

